# Enhancing Gas Permeation Properties of Pebax^®^ 1657 Membranes via Polysorbate Nonionic Surfactants Doping

**DOI:** 10.3390/polym12020253

**Published:** 2020-01-21

**Authors:** Paola Bernardo, Gabriele Clarizia

**Affiliations:** Institute on Membrane Technology (ITM-CNR), National Research Council, Via P. Bucci 17C, 87036 Rende (CS), Italy; p.bernardo@itm.cnr.it

**Keywords:** composite membranes, poly(ether-*block*-amide) copolymers, nonionic surfactants, CO_2_ separation

## Abstract

Composite membranes were prepared by co-casting, incorporating two nonionic surfactants in a poly(ether-*block*-amide), Pebax^®^ 1657 up to 50 wt %. These polysorbate nonionic surfactants contain many ethylene oxide units and are very CO_2_-philic agents; thereby, they can be exploited as membrane additives for gas separation involving carbon oxide. Dynamic light scattering analysis proved a higher stability of additionated Pebax^®^ 1657 solutions with respect to those containing only the copolymer. Scanning electron microscopy showed a regular membrane morphology without pores or defects for all investigated samples. If on the one hand the addition of the additive has depressed the mechanical properties, on the other, it has positively influenced the gas transport properties of Pebax^®^ 1657 films. CO_2_ permeability increased up to two or three times after the incorporation of 50 wt % additive in copolymer matrix, while the selectivity was not significantly affected. The effect of temperature on permanent gas transport properties was studied in the range of 15–55 °C.

## 1. Introduction

Membrane processes represent energy-efficient and low-cost solutions for gas separation. An increasing number of membrane systems is installed in a wide range of technologically relevant applications, such as air separation, hydrogen recovery in refineries, recovery of volatile organics from gas streams, and natural gas upgrading [1].

Poly(ethylene oxide) (PEO) or poly(ethylene glycol) (PEG)-based materials have a good affinity for CO_2_ due to the presence of polar ether oxygens and are thus attractive candidates for separations involving CO_2_ [2,3]. Pebax^®^ are a commercial family of elastomeric multiblock poly(ether-*block*-amide) copolymers with interesting properties as membrane materials. These polymers are thermoplastic elastomers, with different degrees of hydrophilicity depending on the type segments and on their relative ratio [4]. Pebax^®^ 1657, a hydrophilic grade containing approximately 40 wt % Polyamide 6 (PA6) and 60 wt % PEO, can be used as a fouling-resistant coating material [5]. It presents good separation factors for the CO_2_/N_2_ gas pair, but its permeability is not appealing [6]. The higher crystallinity of PA6 domains, compared with PA12 ones (due to the higher content of amide groups that promote hydrogen bonding), leads to lower gas permeation rates [7]. Therefore, different studies were aimed at increasing the permeability of Pebax^®^ 1657. Interesting properties were achieved by blending Pebax^®^ 1657 with polyethylene glycol ethers [6,8,9]. Car et al. identified Poly(ethylene glycol) dimethyl ether (PEG-DME) as the more effective in improving the Pebax permeability, without compromising its selectivity [10]. However, long-term tests demonstrated that the ‘mikado-like’ microstructure of the resulting nanostructured membranes was not stable [11]. Dong et al. reported enhanced CO_2_ separation performance for the hydrophobic Pebax^®^ 2533 grade, which is a block copolymer of 80 wt % poly(tetramethylene oxide) and 20 wt % Polyamide 12 (PA12) upon the addition of nonionic surfactants [12]. 

In this work, the Pebax^®^ 1657 polymer matrix was modified by adding two nonionic surfactants, studying the properties of the resulting dense films in gas separation. In particular, the surfactants are two ethoxylated polysorbates (T20 and T80) that contain a hydrophobic alkyl tail and hydrophilic ethylene glycol head groups. The selected additives are widely used in the food and cosmetic industries due to their nontoxicity. These surfactants are expected to bring additional interactions with the carbon dioxide [12]. Gas transport through the films was investigated, and the gas permeation rates were evaluated as function of the additive content, correlating them to the chemical and mechanical properties of these polymer blends. Indeed, establishing the relationships among the composition, structure, and property of the materials is of great significance for designing high-performance membranes.

## 2. Experimental Section

### 2.1. Materials

Pebax^®^ 1657, a block copolymer, composed of 60% polyethyleneoxide (PEO) and 40% of Nylon 6, was received from Arkema, Rho (MI), Italy. Ethanol (99.5%) (EtOH, from VWR, Milano, Italy) was used for dissolving the Pebax in mixture with distilled water. All the reagents were used as received.

**Figure 1:**
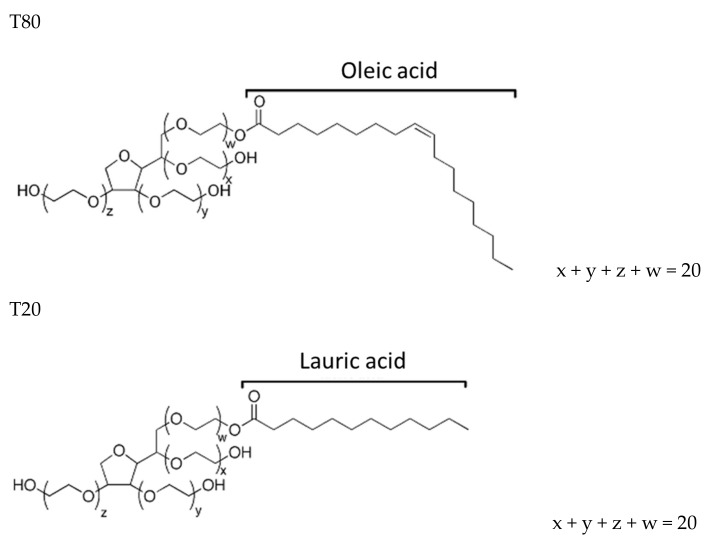
Chemical structure of the polysorbate surfactants.

The polysorbates T20 and T80 (from Sigma Aldrich, Milano, Italy) are nonionic surfactants, ethoxylated sorbital esters of fatty acids with the same PEG content (20 repeat groups of polyoxyethylene units) and a different alkyl tail. T20, a sorbitan monolaurate, presents 12 saturated carbons in the alkyl chain, while the alkyl chain of T80, a sorbitan monooleate, possesses 18 carbons and is unsaturated. These surfactants are soluble in water, with a high value of hydrophilic-lipophilic balance (HLB). Their molecular structure is shown in Figure 1, and their relevant properties are summarized in Table 1. 

The gases for the permeation tests (helium, hydrogen, methane, nitrogen, oxygen, and carbon dioxide), all with purity of 99.99+%, were supplied by SAPIO, Monza, Italy.

### 2.2. Methods

#### 2.2.1. Membrane Preparation

Pebax^®^ 1657 pellets were dissolved in a mixture of distilled water and ethanol (ratio 30:70 wt/wt) at a concentration of 3 wt % under reflux conditions for at least 2 h. Different amounts of the surfactants were added to the Pebax solution. The dope was poured within a steel ring onto a glass plate, leaving the cast films at room conditions overnight, covering the ring in order to reduce the solvent evaporation rate. Dense films were prepared by controlled solvent evaporation with 15, 35 and 50 wt % of surfactants. The films were dried for a few hours at 50 °C.

#### 2.2.2. Membrane Characterization

The size of the aggregates in the polymer solutions was measured by dynamic light scattering (DLS), using a ZetaSizer NanoZS (Malvern Instrument, Malvern, UK). The measurements were made at 25 °C. The Z-average diameters were obtained from the autocorrelation function using a refractive index of 1.51 and 1.35 for polymer and solvent, respectively.

The surface topography of the membranes was studied using an EVO|MA 10 (Zeiss, Milano, Italy) scanning electron microscope (SEM) at an acceleration voltage of 20 kV. Samples were coated with gold using a sputter-coater.

Functional groups of dry dense films were investigated by Fourier Transform Infrared Spectroscopy (FT-IR) analysis in the transmission mode from 4000 to 650 cm^−1^, with a resolution of 4 cm^−1^ averaging 10 scans (FT-IR Spectrum One, Perkin Elmer, Milano, Italy).

Tensile testing of the blend films was performed at room temperature using a single column testing machine (Z2.5, Zwick/Roell, Genova, Italy), having a 50 N load cell and pneumatic clamps. The crosshead speed was 10 mm/min, and the grip separation distance was 20 mm. The average value and the standard deviation of the Young’s modulus, the break strength, and the maximum deformation were determined on a series of at least seven samples having a length of 10 cm and a width of 0.5 cm. 

#### 2.2.3. Gas Permeation Tests

Gas permeation rates were measured on the prepared flat dense membranes in a fixed volume/pressure increase instrument described elsewhere [13]. Before each test, the membrane samples were carefully evacuated with a series of membrane and turbo molecular pumps to remove previously dissolved species. Circular membrane samples with an effective area of 11.3 or 2.14 cm^2^ were used. Their thickness was measured with a digital micrometer (Series 293, Mitutoyo, Lainate MI), Italy), averaging multiple measurements. The tests were conducted at a feed pressure of 1 bar and 25 °C with single gases (H_2_, He, N_2_, O_2_, CH_4_, and CO_2_).

The membrane was exposed to the feed gas, and the pressure in the fixed permeate volume was monitored by a pressure transducer. The slope of the time-pressure curve, considering steady-state conditions, provided the Permeability coefficient (*P)* as described in [13].

The ideal selectivity for two gases (α*_ij_*) was obtained as the ratio of their individual permeability values:

*α*_ij_ = *Pi*/*Pj*. (1)

## 3. Results and Discussion

### 3.1. Solution Stability

**Figure 2:**
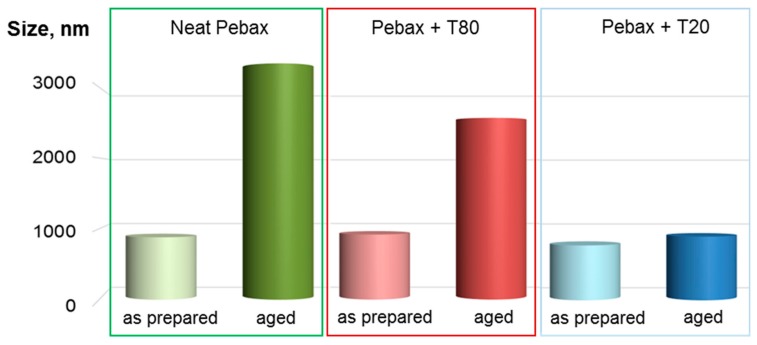
Average size of the aggregates in the Pebax^®^ 1657 (control) and Pebax^®^ 1657/polysorbate 50/50 solutions determined via dynamic light scattering (DLS) analysis.

Typically, Pebax^®^ 1657 solutions tend to form aggregates upon aging under static conditions, becoming a gel [14]. DLS analysis evidenced aggregates in dilute solutions of the neat polymer, with an increasing dimension upon aging. Figure 2 compares a fresh solution with a polymer solution left one day under static conditions. However, the surfactant addition to the polymer solutions resulted in a reduced size of these aggregates. Therefore, the polysorbates tend to stabilize the Pebax^®^ 1657 solutions. Indeed, the surfactant could interrupt polymer chain entanglements in the casting solution. This occurrence was considered as the cause for observed larger pores in ultrafiltration membranes based on poly(l-lactic acid) when using T80 as additive to the polymer dope solution [15]. In the present study, this effect is particularly evident for T20, which is more compatible with the PA6 and PEO segments in the polymer, as can be verified by comparing the Hansen solubility parameters reported in Table 2.

### 3.2. Membrane Morphology

**Figure 3:**
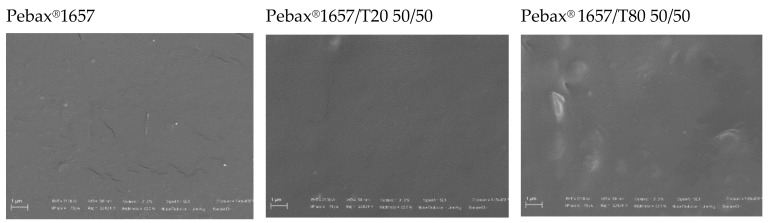
SEM images of the surface of Pebax^®^ 1657 and Pebax^®^ 1657/polysorbate films.

The prepared membranes were easily removed from the glass plate. The films were transparent and with a thickness of ca. 50 micron. Figure 3 displays the surface of Pebax^®^ 1657 and Pebax^®^ 1657/polysorbate membranes at a 50% loading. SEM micrographs evidenced uniform and regular film surfaces without pores or defects. Morphology changes are more distinguishable for the composite films with T80 that had a rougher surface than pure Pebax membrane or Pebax^®^ 1657/T20 samples. Indeed, as discussed before, T20 has a larger affinity with the copolymer blocks. This can be related to the combination of a hydrophilic polymer as Pebax^®^ 1657 with the more hydrophobic surfactant (T80). Indeed, the HLB parameter is equal to 15.0 for T80 and to 16.7 for T20 [21].

### 3.3. Membrane Spectroscopic Properties

**Figure 4:**
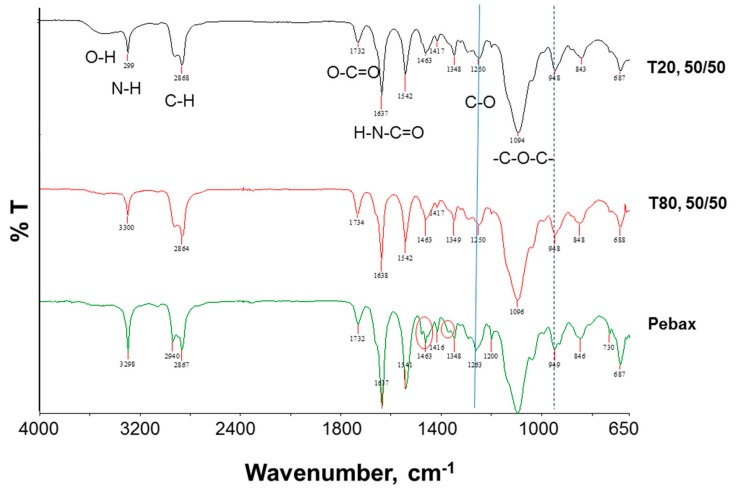
FT-IR (ATR mode) spectra of pristine Pebax^®^ 1657 membrane (control) and Pebax^®^ 1657/Polysorbate membranes.

Figure 4 shows the FT-IR spectra of Pebax^®^ 1657 and Pebax^®^ 1657/Polysorbate membranes.

The spectra taken on neat Pebax^®^ 1657 displayed strong bands at ca. 1100, 1640, and 3300 cm^−1^, assigned to the C–O–C stretching vibrations within the PEO segment, the H–N–C=O stretching vibrations, and the N–H– stretching vibrations in the polyamide block, respectively [22]. The peak at 1732 cm^−1^ is attributed to the –O–C=O groups of the polyamide block in Pebax^®^ 1657 [22]. Typically, polysorbates display a broad and strong band from 3100 to 3500 cm^−1^ related to the O–H stretching vibrations [12]. This is particularly evident in the membrane containing T80.

Compared with the spectra of neat Pebax^®^ 1657 and polysorbates [12], no new absorption peak could be observed in the spectra of Pebax^®^ 1657/polysorbate membranes, suggesting that the additives were physically blended within the polymer matrix. Red shifting in the characteristic peak at 1263 cm^−1^ (C–O bond) can be noticed upon the addition of both polysorbates, indicating H-bond interactions between the soft block in Pebax^®^ 1657 and polysorbates. The bands in the regions of 1270–1350 and 1440–1490 cm^−1^ are indicative of different amide conformations [23]. In the neat Pebax^®^ 1657, the bands at 1348 cm^−^^1^ and 1463 cm^−^^1^ presented a shoulder at higher frequencies (free and bonded). In the blends, the same bands became single, losing the free part, indicating a hydrogen bonded conformation for the polyamide segments. Therefore, both Pebax^®^ 1657 blocks interact with the selected additives.

### 3.4. Membrane Mechanical Properties

**Figure 5:**
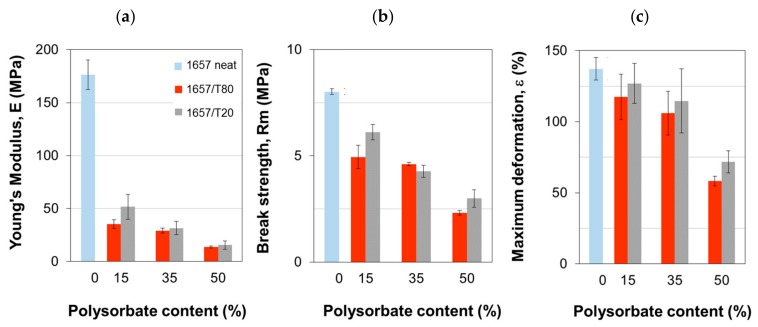
Mechanical properties of the Pebax^®^ 1657 and Pebax^®^ 1657/polysorbate films. (**a**) Young’s Modulus; (**b**) break strength; (**c**) maximum deformation.

The tensile properties of the films are shown in Figure 5. In the Pebax^®^ 1657/polysorbate membranes, the Young’s modulus, the tensile strength, and the elongation at break were depressed with respect to the neat polymer, particularly adding T80. This is due to the polymer dilution in the composite blends. However, the films displayed a stable behavior, while hybrid membranes having inorganic loadings larger than 40 wt % typically present a mechanical failure [24]. In our study, the elastic modulus at the highest surfactant loading (i.e., 16 MPa for Pebax^®^ 1657/T80 and 14 MPa for Pebax^®^ 1657/T20, respectively) reached values even larger than those characteristic of some elastomeric materials. For comparison, the Young’s modulus of polydimethylsiloxane (PDMS) elastomers is reported in the range of 1.32–2.97 MPa, depending on the curing temperature [25].

### 3.5. Membrane Gas Transport

The gas transport properties of the Pebax/Polysorbate membranes, measured at 25 °C, are reported in the following tables. The data of the neat Pebax^®^ 1657 are provided as well. In agreement with the SEM observations, the gas permeation tests indicated the absence of defects in all the membranes and, thus, a good compatibility between both polysorbates and the copolymer. 

The addition of both surfactants to the polymer matrix increased monotonously the gas permeability with respect to the neat polymer (Table 3). CO_2_ has a permeability larger than other smaller gases such as H_2_ and He. Therefore, the prepared films display a reverse-selective behavior [26]. The permeation order of the different gases remained the same as that observed in the neat polymer. However, in the films loaded with T80, methane became more permeable than helium at high loadings.

The ideal selectivity of the prepared membranes is gathered in Table 4. Due to the polar nature of the membrane constituents, improved properties are provided for separating polar and non-polar gas pairs. In general, the ideal selectivity was only slightly depressed. In any case, the ideal selectivity values in the present work were higher than those measured in the reference [12]. Indeed, the Pebax^®^ 1657 matrix is more selective than the Pebax^®^ 2533 matrix.

At the highest additive concentration reached in the blends (50 wt %), depending on the gas species, the Pebax^®^ 1657/Polysorbate membranes presented a two to threefold greater permeability, combined with a modest reduction of the ideal selectivity. T80 was more effective than T20 in increasing the gas permeability, but the latter was more appropriate to keep the original ideal selectivity for different gas pairs (see Table 4), also at high additive concentration. 

**Figure 6:**
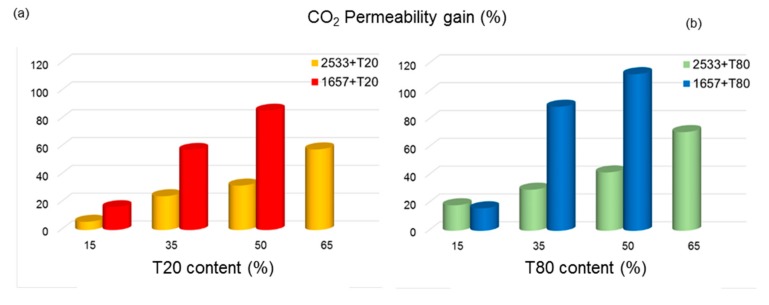
Permeability gain for CO_2_ in the case of membranes doped with T20 (**a**) and T80 (**b**) with respect to the neat polymer: Pebax^®^ 1657 (this work) compared to Pebax^®^ 2533 (from ref. [12]).

Figure 6 shows the relative increase in CO_2_ permeability versus the surfactant content, proposing a comparison with the membranes based on Pebax^®^ 2533 reported by Dong et al. [12]. The neat Pebax^®^ 2533 polymer grade is more permeable than the Pebax^®^ 1657 (e.g., for CO_2_, the paper [12] reports a permeability of 169 Barrer in Pebax^®^ 2533). The greater content of PA6 domains in Pebax^®^ 1657 results in a higher crystallinity of PA blocks if compared with PA12 in Pebax^®^ 2533 owing to the hydrogen bonding promoted by amide groups within the Pebax matrix. Accordingly, the neat Pebax^®^ 1657 has a lower permeability. 

The addition of both polysorbates caused a CO_2_ percentage increase in Pebax^®^ 1657-based samples higher than in literature Pebax^®^ 2533-based films, especially at high additive content. In fact, at a 50 wt % loading of T20, the gain permeability for CO_2_ was 30% [12], whereas in this work, its addition to Pebax^®^ 1657 produced an increase in permeability for CO_2_ of ca. 120%. On the other hand, a concentration of 50 wt % of T80 produced an enhancement in CO_2_ permeability of 40% in Pebax^®^ 2533 [12] and ca. 150% in Pebax^®^ 1657-based films. A larger percentage increment for CO_2_ permeability in the case of Pebax^®^ 1657 can be achieved using lower amounts of the polysorbates, particularly in the case of T80. For this reason, considering the results obtained from mechanical testing, we did not increased the concentration of the additives over 50%. Permeability, which represents the membrane productivity, is even more critical than selectivity in industrial applications [27].

The most permeable sample (Pebax^®^ 1657/T80 (50/50)) stored under ambient conditions was tested after 20 months from the preparation (Table 5), demonstrating a minor decline in permeability (−9.5% for CO_2_) and slightly better selectivities, thus a stable behavior.

**Figure 7:**
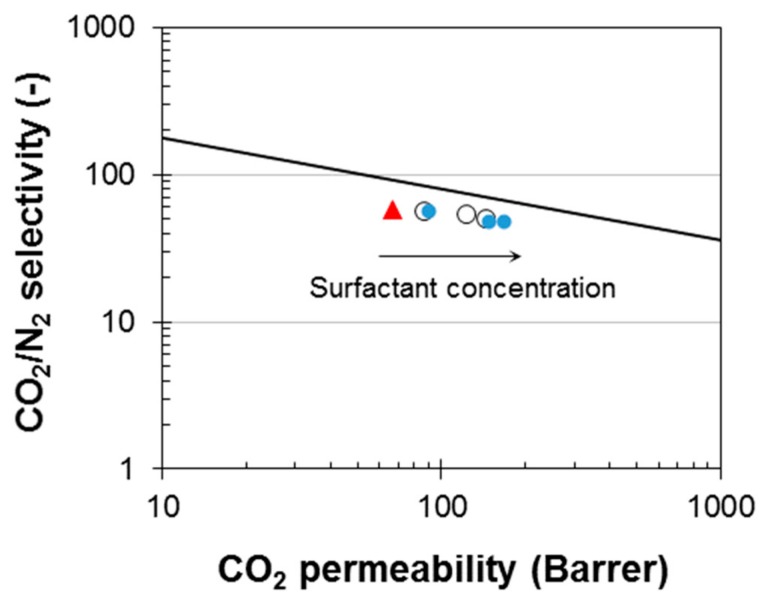
Robeson’s plot for the CO_2_/N_2_ pair showing the data of the prepared membranes. Red triangle (neat Pebax^®^ 1657); open circles (Pebax/T20); closed circles (Pebax/T80). The black line represents the 2008 upper bound [28]. The arrow indicates the increasing concentration of the surfactants in the prepared membranes.

Considering the CO_2_/N_2_ separation, which is relevant for CO_2_ capture from flue gas, the addition of the polysorbates resulted in a slight decline in CO_2_/N_2_ selectivity, which was more visible in the case of T80. A Robeson’s plot for the CO_2_/N_2_ gas pair (Figure 7) shows as the surfactant concentration in the blend membranes increased, their performance moved almost horizontally toward higher CO_2_ permeability, approaching the upper bound [28].

**Figure 8:**
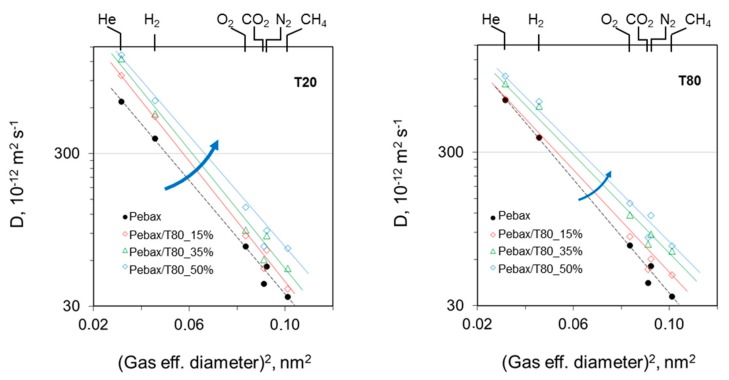
Correlation of the diffusion coefficients with the squared gas molecular size. The arrows indicate the increase in polysorbate concentration.

Assuming the solution-diffusion model, valid for dense isotropic films, according to which permeability can be distinguished in terms of diffusion and solubility, the effect of the additives on both parameters was analyzed. In neat Pebax^®^ 1657, small gas species as H_2_ and He are characterized by higher diffusion coefficients than large molecules as N_2_ and CO_2_. This trend remains unaltered after the addition of T20 and T80, but the absolute value of their diffusion coefficients changes. On the contrary, the solubility remains almost constant except for CO_2_ favored by the presence of specific polar groups in the additive units. The diffusion experimental data correlate well with the squared effective diameter of the gas permeants [29], showing a linear decreasing trend: the larger the molecules, the smaller was the diffusion coefficient (Figure 8). Clearly, the higher the surfactant loading, the higher the diffusion values, owing to a more flexible structure in blend samples. The diffusion selectivity, as expressed by the slope of the lines, was slightly depressed in the membranes loaded with T80, while T20 keeps a similar behavior to that of the neat polymer.

### 3.6. Temperature Effect

A further comprehension of the permeation behavior can be obtained by analyzing the temperature effect on the gas transport. The gas permeability of all tested films increased with temperature, indicating a diffusion-dominated gas transport mechanism in the investigated temperature range. In particular, the temperature dependency of permeability (*P*) can be described by an Arrhenius expression:

*P* = *P*_0_ exp(−*E*_P_/*RT*) (2)

where *P*_0_ is a pre-exponential factor, *E*_P_ is the apparent activation energy for permeation, *R* is the universal gas constant and *T* the absolute temperature [30].

**Figure 9:**
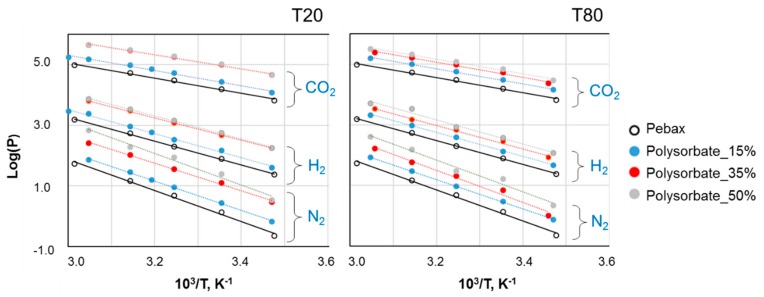
Permeability logarithm versus reciprocal of absolute temperature plot for CO_2_, H_2_, and N_2_.

*E*_P_ can be graphically obtained from the slope of the permeability logarithm versus the reciprocal of absolute temperature plot, as represented in Figure 9 for CO_2_, H_2_, and N_2_.

The order of *E*_P_ was the following: N_2_ ≈ CH_4_ > O_2_ > H_2_ ≈ He > CO_2_, indicating the greatest influence of temperature on the less permeable species (e.g., N_2_) and the lowest effect on CO_2_. In general, the bigger the gas molecule, the larger the resistance experienced for permeation. The only exception is represented by CO_2_, owing to the superior solubility in the pristine material as well as in the blends. Considering that the permeation in polymeric materials follows the solution-diffusion model and that the two processes are affected by temperature in opposite way, low *E*_P_ values depend on a more important role of solubility in the transport. In fact, gas solubility decreases with temperature, while diffusion increases. Typically, the diffusion role is predominant on permeation, and a global increase of permeability with temperature is observed. For species as CO_2_, which is strongly sorbed in the polymer matrix, solubility results are two or three orders of magnitude higher than those for other gases, and the corresponding *E*_P_ is lower. Consequently, the ideal selectivity for fast/slow gases is generally depressed at higher temperature, since the less permeable species have higher activation energy than more permeable molecules.

Despite an increase of the gas permeability in the whole temperature range, a similar response to a temperature change for all gases was observed upon the addition of polysorbate. In fact, the activation energy of the blend samples is close to that of the neat polymer. This is in contrast with the trend shown by other systems, where more permeable materials are characterized by less important activation energy [31].

These findings show the possibility to tailor the properties of the Pebax copolymers by incorporating different amounts of nonionic surfactants (T20 and T80). T80, having a longer alkyl tail than T20, had a pronounced influence on the polymer gas transport properties, inducing a more open and permeable microstructure. This is consistent with the observed morphology in the Pebax/T80 blends (see Figure 3). 

## 4. Conclusions

Composite membranes were successfully prepared by controlled solvent evaporation, by adding nonionic surfactants to the casting Pebax^®^ 1657 solution up to 50 wt %. The additives (polyoxyethylene sorbitan fatty acid esters, T20 and T80) contain ethylene oxide units that are CO_2_-philic agents. Attractive aspects of the prepared blends are related to the green solvent (ethanol) and to the non-toxic and biocompatible surfactants chosen.

The polysorbates are capable of stabilizing the polymer solution against aggregation and gelation, interrupting polymer entanglement. Therefore, they modify the polymer microstructure in the films, as confirmed by morphological analysis. The spectroscopic analysis indicates the establishment of specific polymer/additive interactions. The composite self-standing films display a stable mechanical behavior with lowered tensile properties with respect to the neat polymer. The polysorbate addition results in a steadily enhanced gas permeability, combined to a substantial maintenance of the ideal selectivity. T80 is more effective in increasing the permeability for all gases tested than T20. The films loaded with T80 beneficially exploit the longer hydrophobic alkyl tail of the surfactant and the lower hydrophilic character, while T20 is more affine to the copolymer and is more effective in preserving the dope stability.

## Figures and Tables

**Table 1:** Physical properties of the polysorbate surfactants used in this study.

Surfactant	Molecular Weight (g/mol)	Density (g/mL)	Appearance
PEG20–sorbitan monooleate (T80)	1310	1.064	Golden-yellow viscous liquid
PEG20–sorbitan monolaurate (T20)	1228	1.105	Clear liquid

**Table 2:** Hansen solubility parameters (HSPs) of Pebax^®^ 1657, T20, T80, ethanol/water mixture, and some gases.

	Solubility Parameter (MPa)^0.5^				
Material	*δ*_D_	*δ*_P_	*δ*_H_	*δ*_t_^a^	Ref.
PA6	17.0	10.6	3.4	20.3	[16]
PEO	17.8	0.56	9.1	20.0	[17]
T20				19.5	[18]
T80	19.9	5.0	6.9	21.7	[19]
Ethanol/water mixt.	15.7	11.0	26.3	32.5	[20]
CO_2_	15.7	6.3	5.7	17.9	[20]
N_2_	11.9	0	0	11.9	[20]

^a^*δ*_t_, total cohesion (solubility) parameter: *δ*_t_^2^ = *δ*_D_^2^ + *δ*_P_^2^ + *δ*_H_^2^, where *δ*_D_ is the “Dispersion” parameter, *δ*_P_ is the “Polar” parameter and *δ*_H_ is the “Hydrogen bonding” parameter.

**Table 3:** Pure gas permeability on neat Pebax^®^ 1657 and Pebax^®^ 1657/Polysorbate dense membranes.

Membrane	Additive (wt %)	Permeability (Barrer)					
		H_2_	He	O_2_	N_2_	CH_4_	CO_2_
Neat Pebax	0	6.46	3.92	2.85	1.15	3.41	66.5
T20	15	8.28	5.21	3.92	1.55	5.50	86.7
	35	12.2	8.50	5.67	2.27	7.71	123
	50	16.7	10.3	8.18	2.84	10.2	144
T80	15	8.94	6.12	4.18	1.59	5.60	90.2
	35	14.6	9.24	7.95	3.13	10.0	149
	50	17.4	10.4	8.76	3.49	12.1	167

1 Barrer = 10^−10^ cm^3^ (STP) cm cm^−2^ cmHg^−1^ s^−1.^

**Table 4:** Ideal selectivity of Pebax^®^ 1657 and Pebax^®^ 1657+ T20 or T80 blend membranes.

Pebax/Polysorbate	Ideal Selectivity (−)			
wt/wt	CO_2_/N_2_	H_2_/N_2_	O_2_/N_2_	CO_2_/H_2_
Neat Pebax	57.8	5.62	2.48	10.3
T20 85/15	55.9	5.34	2.53	10.5
T20 65/35	54.3	5.36	2.50	10.1
T20 50/50	50.7	5.88	2.88	8.62
T80 85/15	56.7	5.62	2.63	10.1
T80 65/35	47.6	4.67	2.54	10.2
T80 50/50	47.8	4.99	2.51	9.60

**Table 5:** Permeation data measured on a Pebax^®^ 1657/T80 (50/50 wt %) membrane after an aging time of 20 months.

Permeability (Barrer)						Selectivity (−)		
H_2_	He	O_2_	N_2_	CH_4_	CO_2_	CO_2_/N_2_	O_2_/N_2_	H_2_/N_2_
16.1	9.42	8.21	3.14	11.5	151	48.1	2.61	5.12

1 Barrer = 10^−10^ cm^3^ (STP) cm cm^−2^ cmHg^−1^ s^−1.^
